# Adaptive walking control for quadruped robot by using oscillation patterns

**DOI:** 10.1038/s41598-023-47022-x

**Published:** 2023-11-13

**Authors:** Yong Zhang, Yijia Qian, Yi Ding, Beiping Hou, Rongyang Wang

**Affiliations:** 1 School of Automation and Electrical Engineering, Zhejiang University of Science and Technology, Hangzhou, China; 2 Key Laboratory of Robot System Integration and Intelligent Equipment of Huzhou City, Huzhou, China

**Keywords:** Biophysics, Computational biology and bioinformatics, Engineering, Mathematics and computing, Physics

## Abstract

To improve the adaptability of quadruped robot in multiple scenarios, an adaptive locomotive system based on the double-layered central pattern generator (CPG) is proposed. The novel CPG network consists of double master units and subsets of slave units based on gyroscope signals including yaw and pitch angle. The response of master units provides the ability to control the 1st joins of quadruped robot, while slave units can generate the symmetry signals to control the 2nd and 3rd joints. The CPG network enables the seamless switching of locomotion gaits to stops and starts by using an ultrasonic sensor. Through adjusting the mutually dependent parameters, joints can generate the joint angles to achieve steering behavior. For adaptive movement on an irregular surface, stable ranges of the robot body yaw and pitch angles are proposed by using gyroscope signals. The experimental results verify that the quadruped robot with the proposed double-layered CPG network can perform stable trot pattern in a complex environment.

## Introduction

Quadruped robots have a more remarkable capability to move with diverse movement patterns than wheeled robots especially in unstructured terrain^1^. Numerous outstanding control methods have been developed to control quadruped robot movements such as walking, running and steering^2^. These control methods include model-based control, compliant control and CPG control^3^. Model-based control is the most widely used method in the motion control of legged robots^4^. The calculation of the mechanical characteristics of the robot is an important task^5^. The compliant control method can improve the environmental adaptability based on feedback information^6^. The virtual model controller was proposed to control the quadruped robot as a PID controller^7^. However, researchers have consistently focused on the center of gravity using dynamics equations^8^. When the road conditions change, it is necessary to recalculate the rigid dynamics. With the rapid development of the neural control field, quadruped robots have been studied to utilize neural oscillator techniques to achieve the bionic movement patterns^9^.

In biology, the CPG (central pattern generators) unit located in the spinal cord of vertebrates is able to generate high-dimensional signals for coordinated symmetry movements^10^. The biological CPG network has proved to be a multi-layered structure^11^. Inspired by this method, researchers have developed many important mathematical models to control the quadruped robot^12^. A two-layered CPG network including self-learning function was proposed for the walking motion control of legged robots on irregular terrain^13^. The multi-layered structure allows the biological CPG network to hierarchically process sensory information to control the body parts to improve environmental adaptability^14^.

As for a multi-layered CPG network, researchers have used the properties of higher-order differential equations to generate the periodic signals^15^. Through adjusting the parameters of oscillator units, the CPG network has the ability to generate a stable rhythm signal^16^. However, when the robot moves in a diverse environment, the gait planning becomes single^17^. Thus, getting the adaptive values of mutually dependent parameters is an important work in CPG networks^18,19^. Researchers have proposed to use feedback information to adjust the joints angles for a diversity of environments^20–22^.

Diversified movement patterns are the most effective in unstructured environments^23^. The trot movement pattern is a gait plan in which the diagonal legs pair up and hit down at separate intervals in animals such as the horse^24^. Quadruped robots use the trot pattern to control the center of gravity easily^25^. Because the double-layered CPG network consists of higher order differential equations, it is easy to integrate abundant sensing signals^26^. Through information from the environment, CPG networks have appropriate characteristics for motion control under the trot pattern. This kind of controller has the ability to achieve adaptive motion in the quadruped robot in multiple scenarios^27^.

In this paper, an adaptive locomotive system based on the double-layered central pattern generator (CPG) is proposed, to improve the adaptability of the quadruped robot in multiple scenarios. The novel CPG network consists of double master units and subsets of slave units. The response of master units renders them the ability to control the 1st joins of quadruped robot, while slave units can generate the symmetry signals to control the 2nd and 3rd joints. By implementing this concept, the CPG networks of central pattern generators feature the capability to generate the symmetry signals for stable movement and steering under the trot pattern. For adaptive motion on an irregular surface, a stable range of the robot body yaw and pitch angles are proposed based on gyroscope signals. The CPG network enables the seamless switching of locomotion gaits to stops and starts based on an ultrasonic sensor. The experimental results verify that the quadruped robot with the proposed double-layered CPG network can perform stable trotting in a complex environment.

The rest of the paper is organized as follows. In “Quadruped robot model” section, the model of quadruped robot is presented and the contribution of different body parameters are discussed. “Model of CPG unit” section discusses the central pattern generator and formulates a trajectory generator based on different mutually dependent parameters. In “Model of CPG network” section, the network of this control system is discussed and verified. In “Experiment” section, the results are further evaluated and compared with the experimental results. In “Conclusions” section the conclusions are presented.

## Quadruped robot model

**Figure 1:**
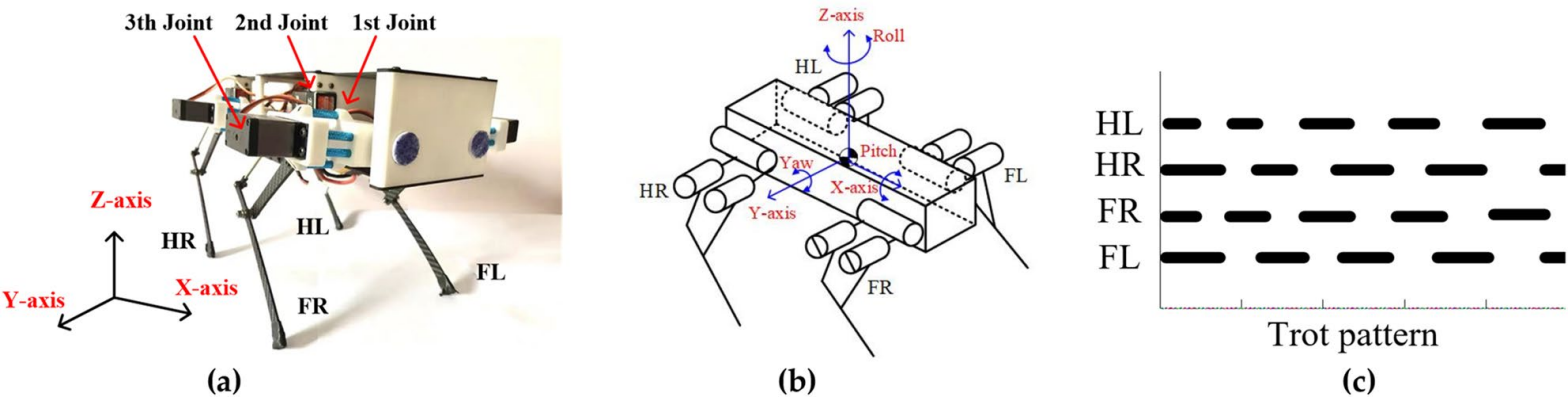
The quadruped robot platform: (**a**) mechanical structure of the quadruped robot; (**b**) structural sketch of robot; (**c**) the trot gait.

Figure 1a,b present the appearance of the quadruped robot named Standford Pupper, which controlled by 12 degrees of freedom. Each leg has three active joints that provide a total of three degree of freedom: first pitch joint, second yaw joint and third yaw joint. The second and third joints mostly provide the primary power to the forward movement of the robot. These joints are driven by a steering actuator. The servomotor of the robot is Servo CL S6336HV. The communication among the servo motors is serial communication bus. To recognize obstacles during robot movement, an ultrasonic sensor is installed on the body of the robot. To measure the body angle, a gyroscope sensor is equipped inside of the body to feedback the yaw, pitch and roll angles. The robot controller installed inside the robot body can generate the signal of each joint. The power is supplied from a battery.

The overall dimensions are 20 cm height, 33 cm length, and 1.65 kg total weight. Due to the small size of robot, the dynamic characteristics will affect the motion stability. The constraint condition of stability is the distances from the projection point of the COG (center of gravity) to the sides of the supporting polygon. The stability margin of the trot gaits is always positive in the moving processes. To design the phase of the trot pattern, the sequence of the leg is defined as Hind-Left (HL) leg → Front-Right (FR) leg → Hind-Right (HR) leg → Front-Left (FL) leg during movement in the trot pattern, as shown in Fig. 1c. Through the joints control with the trot walking pattern, the quadruped robot can suitable for complex environments. This mechanical setup is based on our previous work on the model of Featherstone rigid dynamics^28^. By using this mechanical structure, the quadruped robot has the ability to move with a trot pattern. The physical parameters of the robot are listed in Table 1.

**Table 1:** The dimensions of robot.

	1st joint	2nd joint	3th joint
Length (cm)	5	12	15
Width (cm)	7	4	1.5

## Model of CPG unit

**Figure 2:**
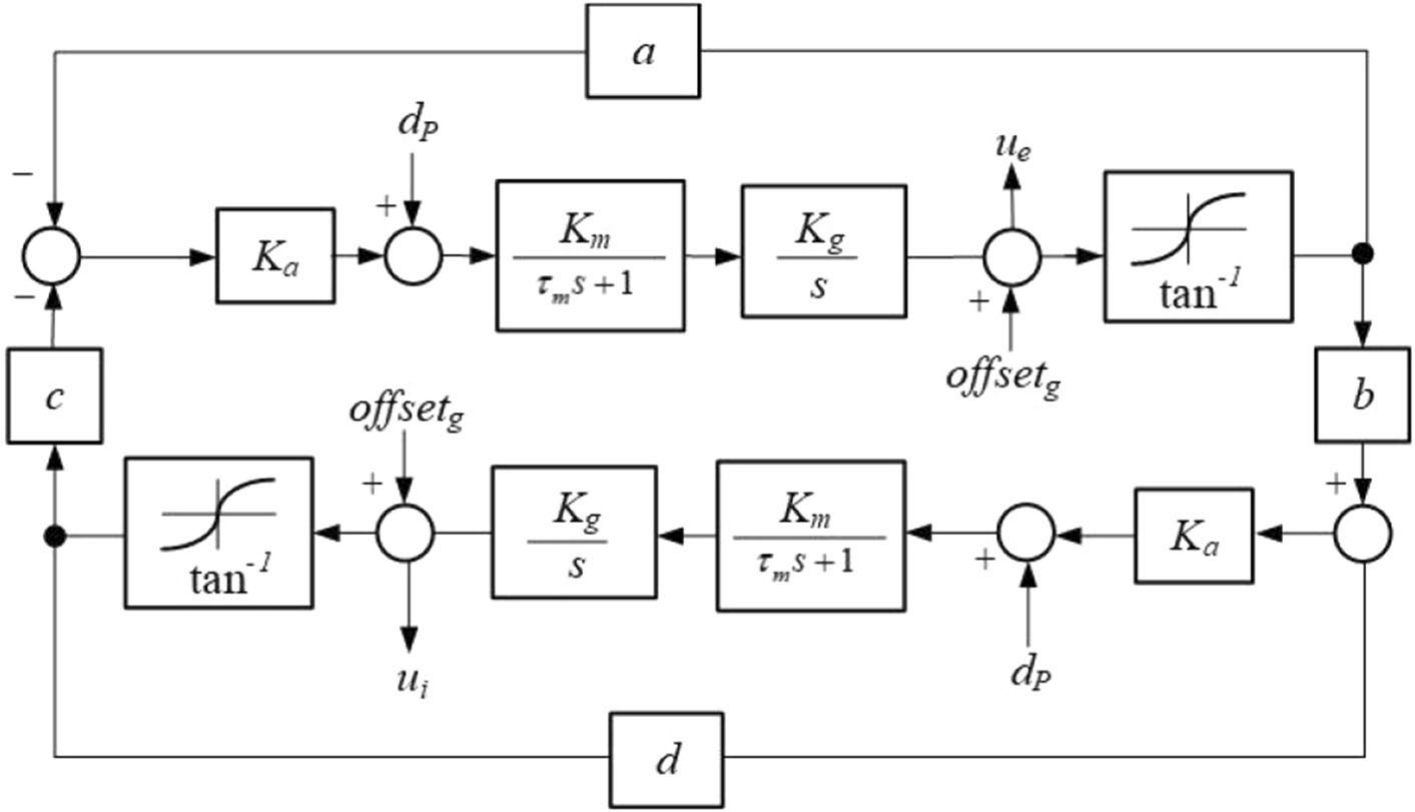
The block diagram of CPG unit.

In order to achieve movement in the trot pattern, the model of CPG unit is proposed. There is an excitatory and an inhibitory unit to generate the periodic signals depending on the coupling parameters. The internal state of the excitatory unit is $u_{e}$ and the inhibitory unit is $u_{i}$. The joint angles are generated by mutually dependent parameters in real time. Figure 2 shows the block diagram of the proposed CPG model.

A function of the dynamic parameters is output as a series of periodic signals for controlling the joint motor, where $a$ represents the intrinsic excitatory coupling parameter, $d$ represents the intrinsic inhibitory coupling parameter, $b$ represents the inhibitory coupling factor and $c$ is the excitatory coupling factor. To control the joint angle directly, the motor dynamic parameters are used. $K_{m}$ represents the gain constant of the motor, $\tau_{m}$ represents the time constant, $K_{g}$ represents the gear ratio, and $K_{a}$ represents the gain of the motor amplifier. Based on the dynamic parameters of the motor, the model of CPG unit can generate the periodic signals in the robot joints directly. The parameter sets are shown in Table 2. The dynamic equations of the proposed CPG model are formulated as follows:

1 $$ \begin{aligned} \tau_{m} \frac{{d^{2} u_{e} }}{{dt^{2} }} & = - \frac{{du_{e} }}{dt} - offset_{g} - K_{g} K_{m} K_{a} a\tan^{ - 1} u_{e} + K_{g} K_{m} d_{P} \\ & \quad - K_{g} K_{m} K_{a} c\tan^{ - 1} u_{i} + d\tan^{ - 1} u_{e} \\ \tau_{m} \frac{{d^{2} u_{i} }}{{dt^{2} }} & = - \frac{{du_{i} }}{dt} - offset_{g} + K_{g} K_{m} K_{a} b\tan^{ - 1} u_{e} + K_{g} K_{m} d_{P} + d\tan^{ - 1} u_{i} \\ offset_{g} & {\text{ = K}}_{r} (\theta - \theta_{o} ) \\ d_{p} & { = }\left\{ {\begin{array}{*{20}c} 1 & {U_{i} \ge {10}} \\ { - \left( {\frac{1}{{\sum\limits_{i = 1}^{n} {\left\| {} \right.U_{i} \left. {} \right\|} }}} \right)^{2} } & {otherwise} \\ \end{array} } \right. \\ \end{aligned} $$

**Table 2:** CPG parameter sets.

Parameters	$a$	$b$	$c$	$d$	$K_{g}$	$K_{a}$	$K_{m}$	$K_{r}$	$K_{u}$
Value	2.1	17.1	18.1	− 18.1	1.6	4.9	120	14	18

The gyroscope can detect the body angle to the CPG model in real time, where $offset_{g}$ represents the parameters to feedback this information, and ${\text{K}}_{r}$ represents the feedback gain. For the first joint angle of the robot, $\theta$ represents the yaw angle measured in real time, and $\theta_{o}$ represents the stable body yaw angle of the robot. For the other joint angles of the robot, $\theta$ represents the pitch angle measured in real time, and $\theta_{o}$ represents the stable body pitch angle of the robot. If the robot approaches an upward or downward inclination road, its yaw and pitch angle changes quickly. The gyroscope will detect this information and feed it back to the CPG model immediately. If the body angle deviates from the stable range, $offset_{g}$ will rise to a high value to control the CPG model to generate a high cyclic period until the body angle returns to the stable range. At this time, $offset_{g}$ becomes the original value again. By means of this concept, the robot can overcome the terrain condition through adaptive rhythmic joint motion.

Here, $d_{P}$ represents feedback parameters for the ultrasonic sensor, $U_{i}$ represents the value of ultrasonic sensor in real time, and $K_{u}$ is the gain constant. Depending on the value of $d_{P}$, the CPG model will be changed to adjust the first joints of robot. If there is an obstacle detected by the ultrasonic sensor, the value of $d_{p}$ will change. The oscillation network can output the signals with higher amplification. The robot will turn and leave the obstacle until no obstacles are ahead. At this time, $d_{p}$ switches to 1 again. The CPG model can continue to generate periodic signals for the forward movement of the robot.

## Model of CPG network

**Figure 3:**
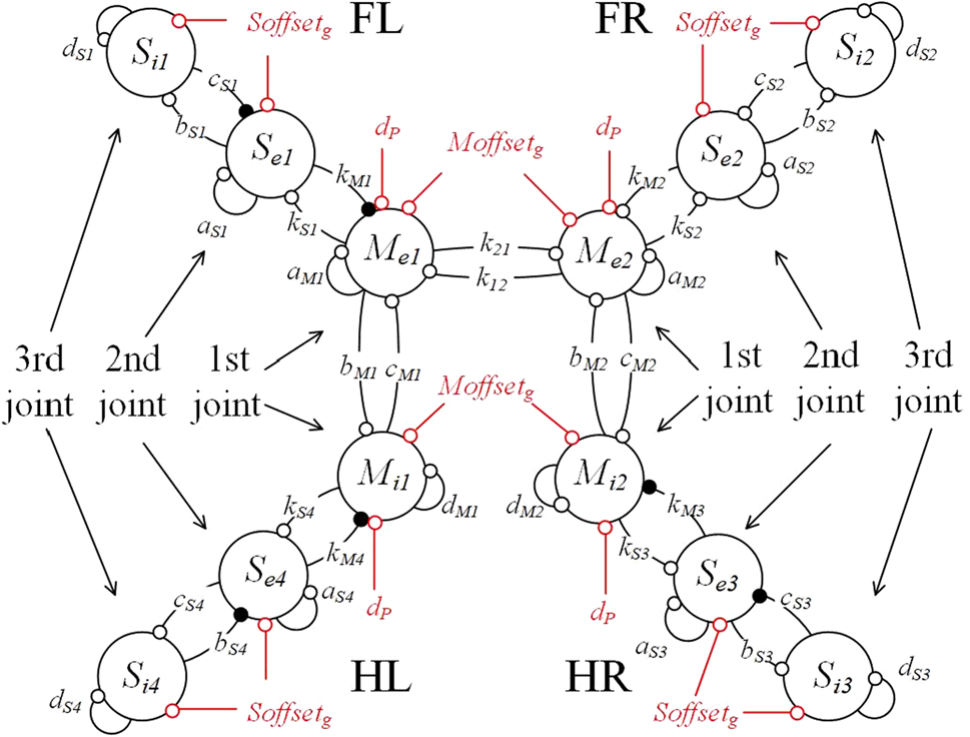
The framework of CPG network.

In order to generate the fundamental gait signals for the quadruped robot, the CPG models are mounted on each leg, as depicted in Fig. 3. Here, FL, FR, HL, and HR denote the Front-Left leg, Front-Right leg, Hind-Left leg and Hind-Right leg, respectively. In this research, the CPG network consists of double master units and subsets of slave units. The response of master units $M_{e1}$, $M_{i1}$, $M_{e2}$ and $M_{i2}$ have the ability to control all the 1st joins. The models are connected to each other by coupling parameters $k_{{{12}}}$ and $k_{{{21}}}$. The parameter $d_{P}$ feedbacks the signals of ultrasonic sensor. $Moffset_{g}$ represents the yaw angles of the robot body.

Slave units can generate the symmetry signals to control all of the 2nd and 3rd joints of the quadruped robot. The mutually dependent parameters, including $$k_{Mj}$$ and $$k_{Sj}$$$$(j = 1 - 4)$$, can be adjusted. $$Soffset_{g}$$ represents the pitch angles of the robot body. Through the mutually dependent parameters, the models of CPG units are connected to control the quadruped robot. The dynamic equations of the CPG network in the Front-Left leg are expressed as follows:

2 $$ \left\{ \begin{aligned} \tau_{m} \frac{{d^{2} u_{Me1} }}{{dt^{2} }} & = - \frac{{du_{Me1} }}{dt} - Moffset_{g} - K_{g} K_{m} K_{a} a_{M1} \tan^{ - 1} u_{Me1} + d_{M1} \tan^{ - 1} u_{Se1} \\ & \quad - K_{g} K_{m} K_{a} c_{M1} \tan^{ - 1} u_{Mi1} + K_{g} K_{m} d_{p} + k_{12} \tan^{ - 1} u_{Me2} \\ \tau_{m} \frac{{d^{2} u_{Mi1} }}{{dt^{2} }} & = - \frac{{du_{Mi1} }}{dt} - Moffset_{g} + K_{g} K_{m} K_{a} b_{M1} \tan^{ - 1} u_{Me1} \\ & \quad + K_{g} K_{m} d_{i1} d_{p} + k_{M4} \tan^{ - 1} u_{Se4} \\ Moffset_{g} & = K_{y} \theta_{yaw} \\ \end{aligned} \right. $$

3 $$ \left\{ \begin{aligned} \tau_{m} \frac{{d^{2} u_{Se1} }}{{dt^{2} }} & = - \frac{{du_{Se1} }}{dt} - Soffset_{g} - K_{g} K_{m} K_{a} a_{S1} \tan^{ - 1} u_{Se1} + d_{S1} \tan^{ - 1} u_{Me1} \\ & \quad - K_{g} K_{m} K_{a} c_{S1} \tan^{ - 1} u_{Si1} + K_{g} K_{m} \\ \tau_{m} \frac{{d^{2} u_{Si1} }}{{dt^{2} }} & = - \frac{{du_{Si1} }}{dt} - Soffset_{g} + K_{g} K_{m} K_{a} b_{S1} \tan^{ - 1} u_{Se1} + K_{g} K_{m} d_{i1} \\ {\text{S}}offset_{g} & = K_{p} \theta_{pitch} \, \\ \end{aligned} \right. $$

**Figure 4:**
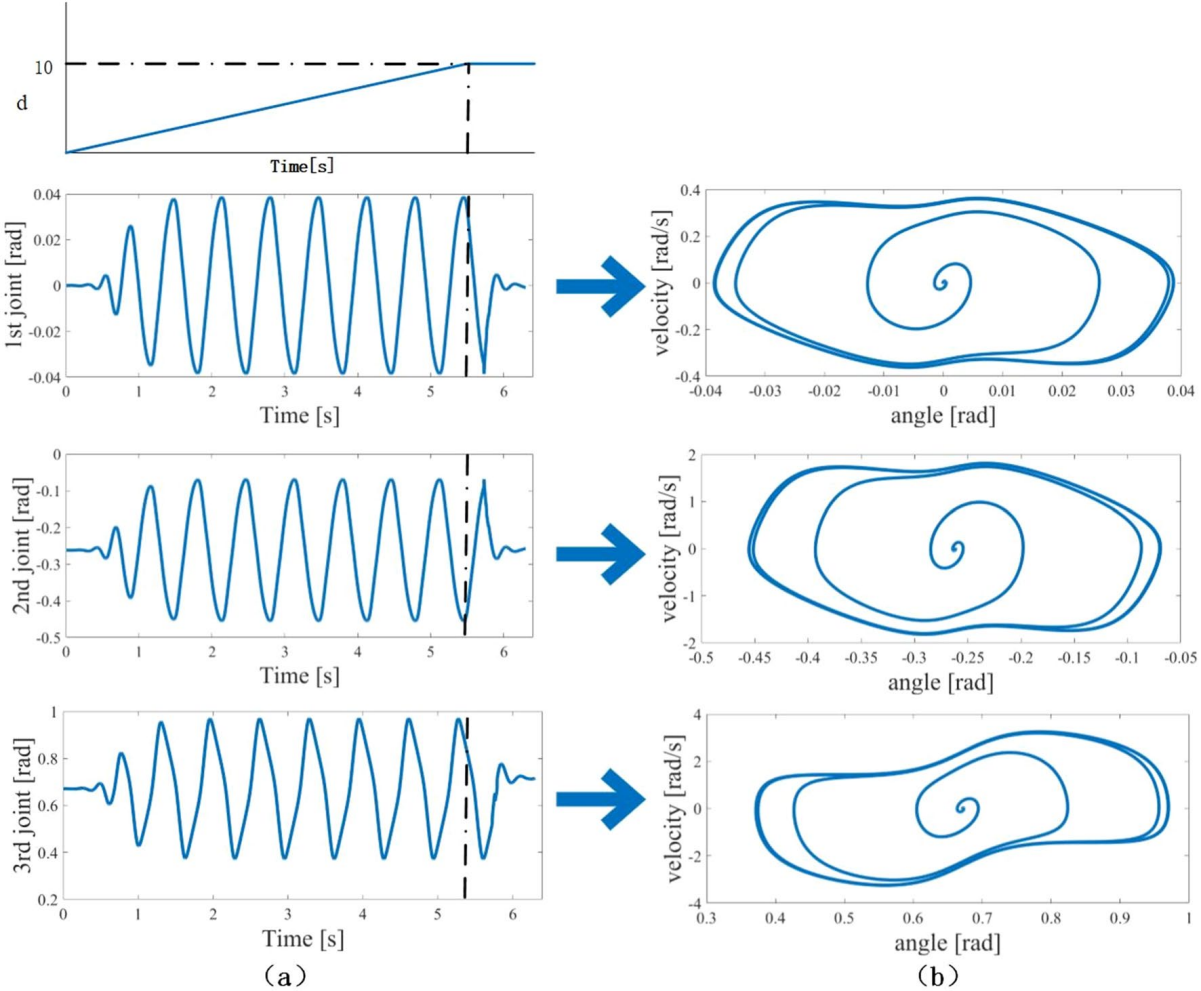
Examples of the phase-coordinated CPG network; the relevant parameters are provided in Table 2. (**a**) The response of CPG network under $d_{P}$ switching; (**b**) Evolution of the limit cycle during the gait switch.

Once the CPG network reaches a stable oscillation state, it generates periodic signals whose shapes depend on the internal parameters, the network topology and interconnection weights, as shown in Fig. 4. The coefficients and limits of each polynomial are found experimentally to yield a good fitting with low-order polynomials.

In order to create the stable periodic signal of the CPG model, a limit cycle is designed that defines a series of exact repetitions with a closed trajectory for the trotting motion of the robot. The trajectory of the limit cycle starts with a fixed point and continues around a nominal orbit swift. Depending on resetting the phase, the oscillation network can generate a series of signals to spin in circles.

The amplifier and frequency are used to control the quadruped robot. The CPG control system generates the joint angles with the trot pattern. Through the ultrasonic sensor, the response of $u_{Me1}$ and $u_{Mi1}$ is based on the adjustment of $d_{p}$. At 5.5s, the value of $d_{P}$ become 10, the CPG network can be updated to adjust gait patterns of output, and the joints stop immediately using the internal feedback signals. By using this way, the CPG network has ability to generate suitable motions.

**Figure 5:**
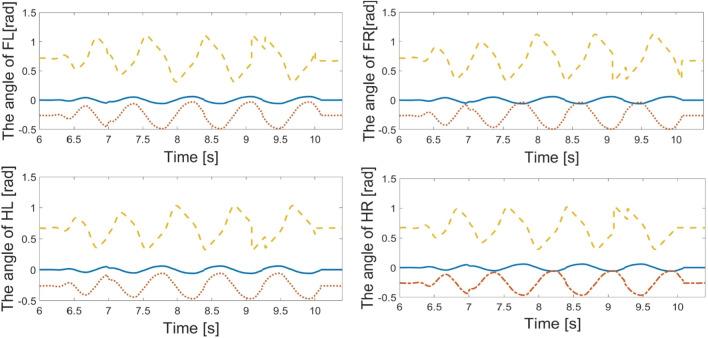
The response of CPG network for steering motion under mutually parameters control. The joints angle of FL, FR, HL, HR legs of robot.

Through setting the mutually dependent parameters, $$k_{{{12}}}$$, $$k_{{{21}}}$$, $$k_{Mj}$$ and $$k_{Sj}$$$$(j = 1 - 4)$$, the joints can generate the joint angles to achieve steering behavior. The angle of steering behavior is $$\pi {/2}$$. Because the robot is steering right with the trot pattern, the amplitudes for the right swing joints are smaller than those for the left swing joints. The diagonal limbs move out of phase, and the generated trajectories must be in phase. The responses are shown in Fig. 5. The first joints, second joints and third joints are depicted in blue, yellow and red lines, respectively. All of the generated trajectories respect the coordination constraints imposed by the couplings.

The phase difference between the output signals is determined by the internal parameters of the neurons, the CPG network topology and the inter-connection weights. These parameters have been experimentally chosen to yield output signals with a shape resembling a periodic wave as much as possible, given the weights and internal parameters. By adjusting the value of feedback parameters, the response of $u_{Me1}$ and $u_{Mi1}$ can reset the amplifier and frequency of signals. By adjusting the value of feedback parameter, the output of oscillation network can generate periodic signals to respond to the external stimuli. The parameters $u_{Me1}$ and $u_{Mi1}$ generate the periodic signals stably by using mutually dependent parameters. The coefficients of the low-order polynomials and the corresponding coefficients of determination are given in Table 3.

**Table 3:** The mutually parameters sets.

	$k_{12}$	$k_{21}$	$k_{M1}$	$k_{S1}$	$k_{M2}$	$k_{S2}$	$k_{M3}$	$k_{S3}$	$k_{M4}$	$k_{S4}$
Walk forward	− 1.8	1.8	1.1	1.15	1.8	− 1.6	− 1.8	1.8	− 1.6	1.6
Steering motion	2.1	2.3	0.8	2	− 1.8	1.6	1.8	− 1	1.6	− 2

## Experiment

In order to confirm the validity of the CPG network, two sets of experiments are conducted for the application of adaptive movement system for quadruped robot control. Firstly, the robot trots forward and steers by using the ultrasonic sensor. Secondly, the robot trots on an irregular surface by using its gyroscope sensor. The controller of the robot is Raspberry Pi. To record the data, wireless modules are installed in the robot body. By using the XBee modules, the controller can send the joint angles to a computer.

**Figure 6:**
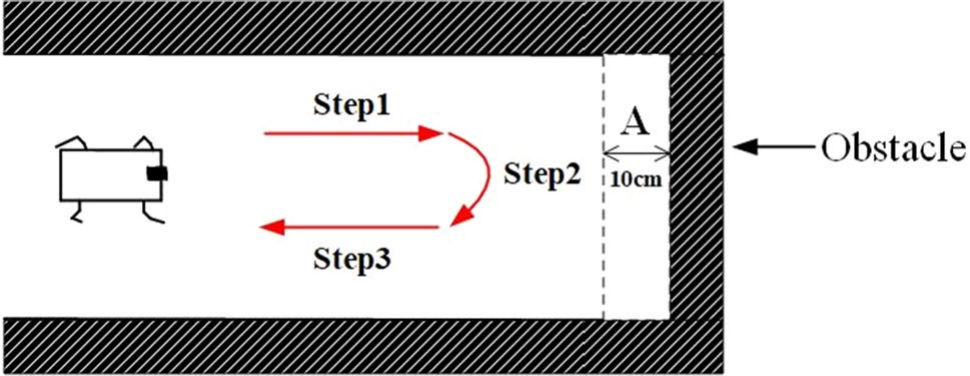
The map of the first experiment.

In the first experiment, the experimental environment is shown in Fig. 6. The distance of region $A$ is set to 10 cm. Such information is unknown to the quadruped robot. The mutually dependent parameters of CPG networks are shown in Table 2. The response of each neuron can generate the robot joint based on the stimuli of feedback parameters. The experimental results demonstrate that the oscillation network can autonomously generate the periodic signals for the quadruped robot.

**Figure 7:**
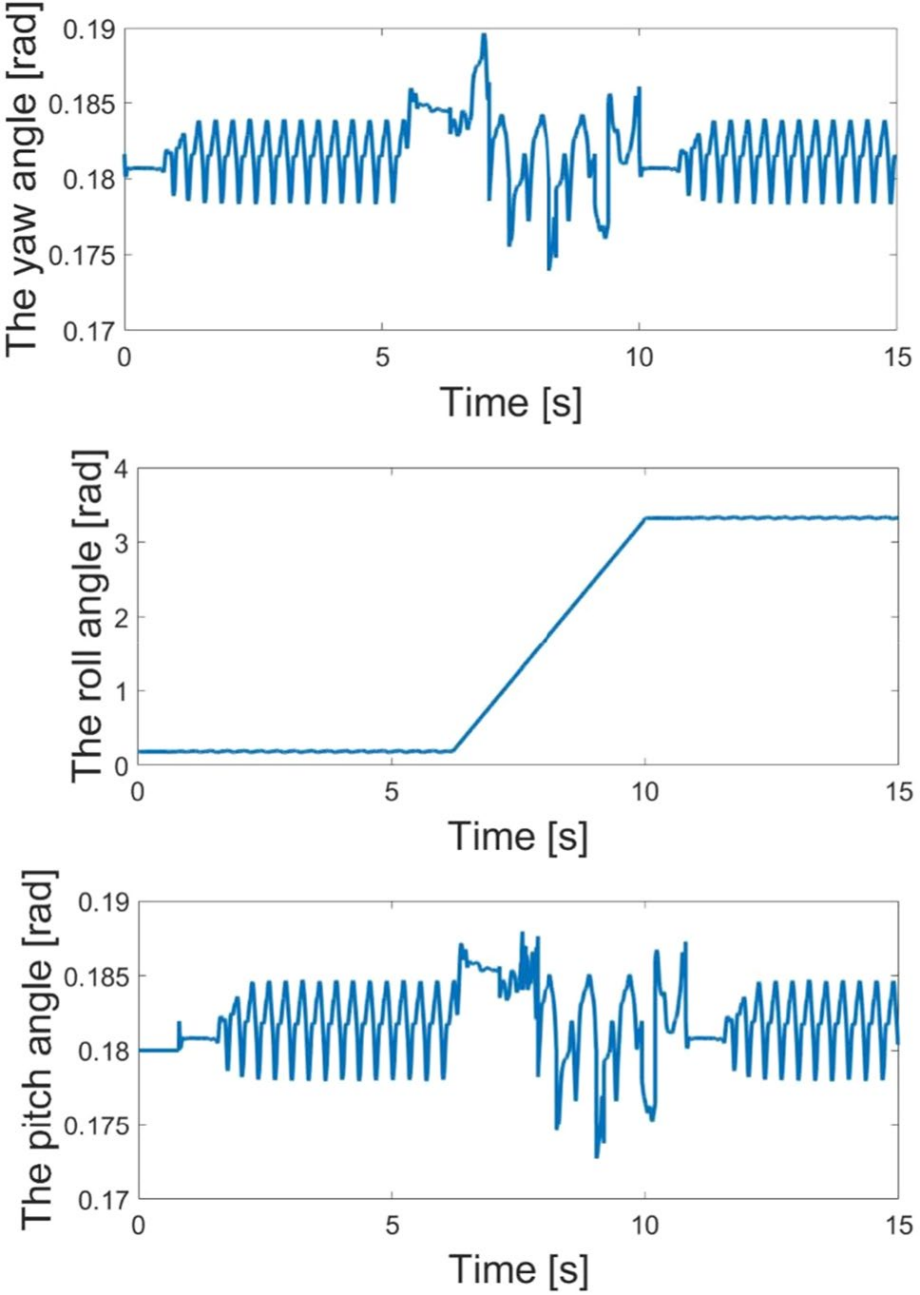
The yaw, roll and pitch angles of robot in the first experiment.

Figure 7 illustrates the body angle along the X axis, Y axis and Z axis during the whole process. Before 6s, the robot moves along the road in a trot pattern. Depending on the CPG network, the speed is stable, and the amplifier and frequency of body angle is regular. The quadruped robot is able to trot stably. However, at 6s, the robot trots into the $A$ area, and the ultrasonic sensor detects the obstacle. The body angles change sharply because of the parameters $d_{p}$ adjusting. At this time, the master and slave oscillation units adjust the mutually dependent parameters. The oscillation networks change the amplifier of robot joints to steer for leaving the narrow space. At 7.5 s, the robot finishes the steering behavior, the ultrasonic sensor detects the distance of obstacle, and robot needs to continue to steer. At 9.8 s, there is no obstacle in front of the quadruped robot and it stops steering. The mutually dependent parameters switch to moving forward. At last, the robot leaves the narrow space in a trot pattern by using CPG network control. The experiment demonstrates that the robot body angles are stable due to the adjustment of feedback parameter including the feedback of ultrasonic sensor.

**Figure 8:**
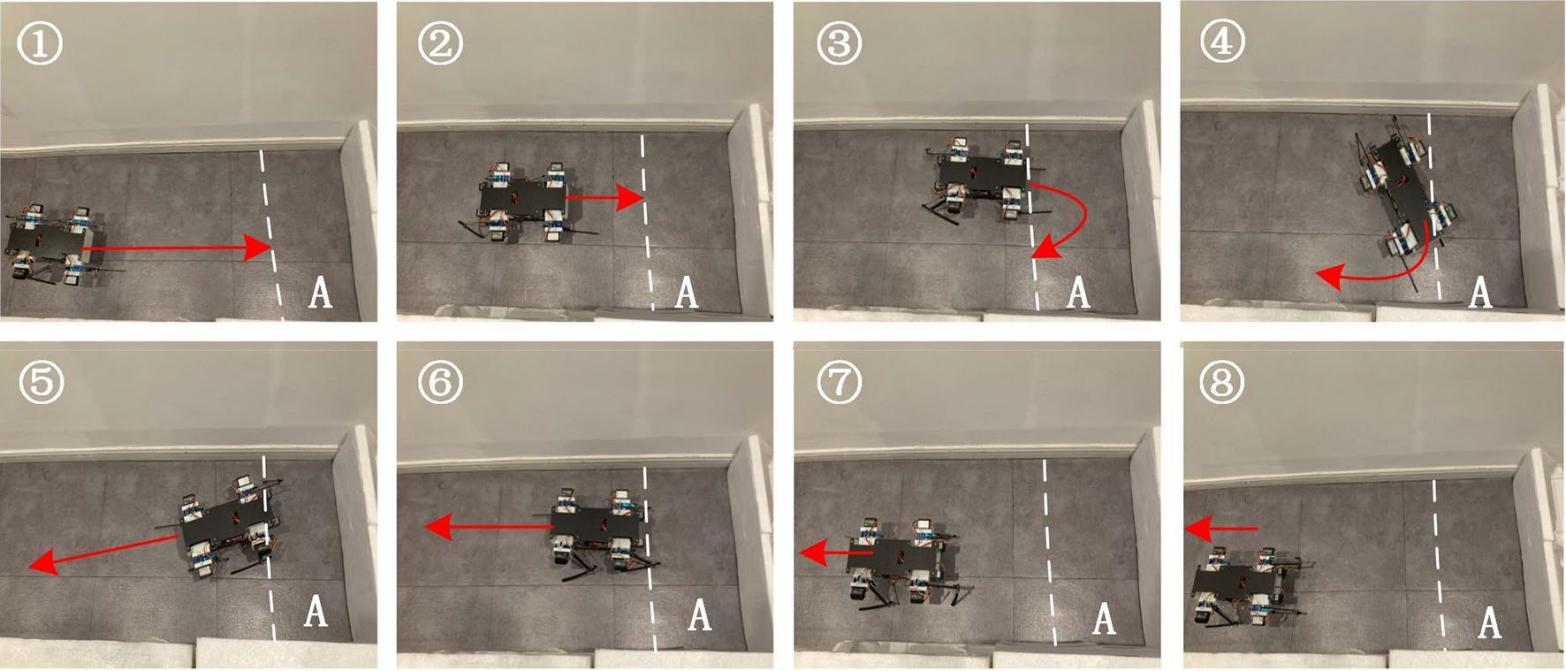
The frame of first experiment.

The scenes of quadruped robot in the whole experiment are shown in Fig. 8. Based on the feedback of ultrasonic sensor, the quadruped robot can move forward and then stop in front of the obstacle. Through adjusting the mutually dependent parameters, the double-layered CPG network can set the response for steering behavior. The quadruped robot can move stably in a trot pattern.

**Figure 9:**
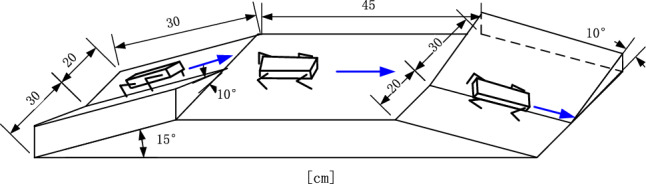
Up-down slope used in experiment.

In the second experiment, the irregular surface includes an up-down slope, as shown in Fig. 9, with the angle of slope at 15 degrees. On the up and downward surface, there is a slope with a 10-degree angle and the robot is unaware of this irregular road condition. Using the gyroscope signals can provide feedback in real time. The range of the pitch and yaw angle are within ± 8 degrees. If the pitch and yaw angle deviates from this range, the CPG network will change the parameters for the slope.

**Figure 10:**
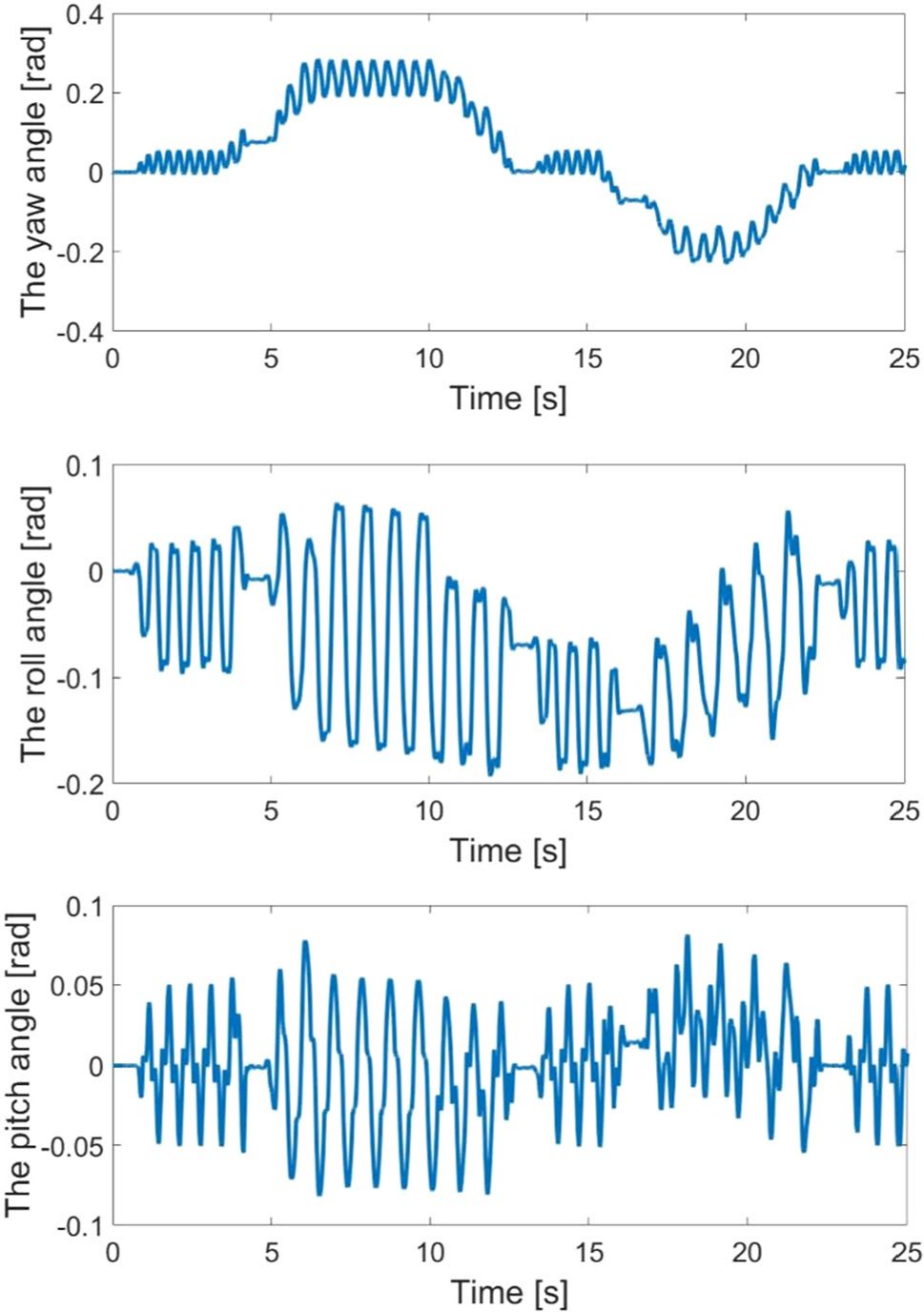
The yaw, roll and pitch angles of robot in the second experiment.

Figure 10 shows the body angle in the X axis, Y axis and Z axis during the whole process. Before 5 s, the quadruped robot moves on the flat surface. Because the pitch and yaw angle do not deviate from the stable range, the robot moves using the parameter set for the flat surface. At 6 s, the robot moves on the upward slope, where the pitch angle deviates from the stable range. The yaw angle also increases suddenly, and the gyroscope detects this information. By changing the parameters of feedback, the first joint angles adjust to keep the pitch angle in a stable range. The second and third joint angles can generate the appropriate periodic joint motions. At 18 s, the robot approaches the down-slope, and the yaw and pitch angles change acutely. At this time, the CPG network generates high cyclic period signals.

**Figure 11:**
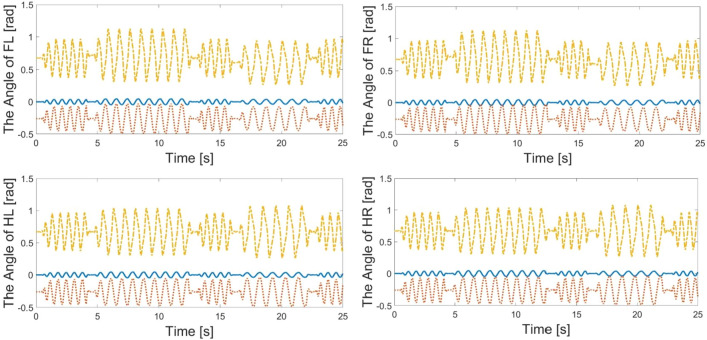
The response of FL, FR, HL, HR in the second experiment.

**Figure 12:**
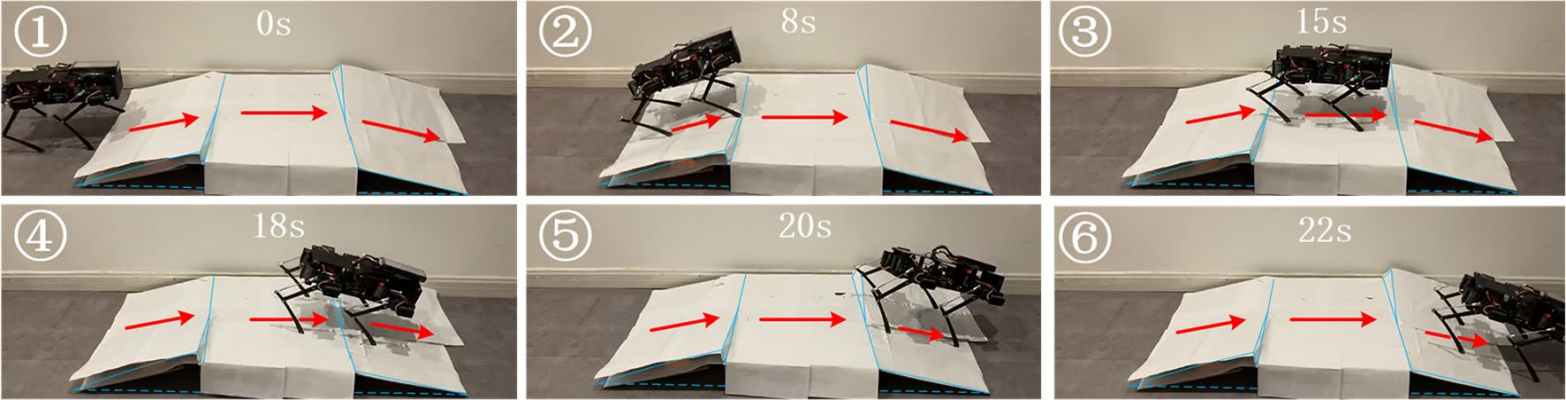
The frame of second experiment.

Figures 11 show the responses of the joint angles. When the robot enters the up-down slope, it detects the change in the road surface and the internal feedback parameters are adjusted automatically. The results for the quadruped robot are shown in Fig. 12.

The above experiments show that the proposed CPG control system can allow the robot to move in a complex environment to avoid abrupt changes, demonstrating improved adaptability in multiple scenarios. This result is crucial for extending the practical applications. The double-layered CPG network can generate the periodic joint angle to control the quadruped robot on the irregular terrain. For adaptive movement on an irregular surface, a stable range of the robot body yaw and pitch angles are proposed based on gyroscope signals.

## Conclusions

In this research, the model of CPG unit including motor dynamics parameters for quadruped robot has been proposed. An adaptive locomotive system based on the double-layered central pattern generator (CPG) introduced to improve the adaptability of the quadruped robot in multiple scenarios. The novel CPG network consists of double master units and subsets of slave units based on gyroscope signals, including yaw and pitch angle. To generate the regular oscillatory signals for the quadruped robot, the master and slave CPG models are mutually connected to each other to control the joints of each leg. In the master unit, by using coupling parameters, the output of each neuron is directly connected to the slave neuron. The response of master units is able to control the 1st joins of the quadruped robot, while the slave units can generate the symmetry signals to control the 2nd and 3rd joints. The response of each neuron can generate the robot joint based on the stimuli of feedback parameters. Through adjusting the mutually dependent parameters of CPG networks, the joint angle can be controlled based on feedback information. For adaptive movement on an irregular surface, a stable range of the robot body pitch and yaw angle are proposed by using gyroscope signals. To detect the obstacle, the ultrasonic signals are utilized for adjusting the parameters. The double-layered CPG networks have a capability to generate the symmetry signals for stable movement on a complex surface.

By using our proposed concept, the quadruped robot can be controlled by double-layered CPG network, while generating the symmetry signals periodically depend on feedback from the information of sensors. This realizes the effective implementation of adaptive motion in the quadruped robot. Two set of experiments confirm the validity of the proposed control system.

## Data Availability

All data generated during this study are included in this published article. The datasets used and analyzed during the current study available from the corresponding author on reasonable request.
